# Extending rule-based methods to model molecular geometry and 3D model resolution

**DOI:** 10.1186/s12918-016-0294-z

**Published:** 2016-08-01

**Authors:** Brittany Hoard, Bruna Jacobson, Kasra Manavi, Lydia Tapia

**Affiliations:** Department of Computer Science, University of New Mexico, Albuquerque, 87131 New Mexico USA

**Keywords:** Rule-based model, Geometric model, Antigen-antibody interactions, Molecular assembly

## Abstract

**Background:**

Computational modeling is an important tool for the study of complex biochemical processes associated with cell signaling networks. However, it is challenging to simulate processes that involve hundreds of large molecules due to the high computational cost of such simulations. Rule-based modeling is a method that can be used to simulate these processes with reasonably low computational cost, but traditional rule-based modeling approaches do not include details of molecular geometry. The incorporation of geometry into biochemical models can more accurately capture details of these processes, and may lead to insights into how geometry affects the products that form. Furthermore, geometric rule-based modeling can be used to complement other computational methods that explicitly represent molecular geometry in order to quantify binding site accessibility and steric effects.

**Results:**

We propose a novel implementation of rule-based modeling that encodes details of molecular geometry into the rules and binding rates. We demonstrate how rules are constructed according to the molecular curvature. We then perform a study of antigen-antibody aggregation using our proposed method. We simulate the binding of antibody complexes to binding regions of the shrimp allergen Pen a 1 using a previously developed 3D rigid-body Monte Carlo simulation, and we analyze the aggregate sizes. Then, using our novel approach, we optimize a rule-based model according to the geometry of the Pen a 1 molecule and the data from the Monte Carlo simulation. We use the distances between the binding regions of Pen a 1 to optimize the rules and binding rates. We perform this procedure for multiple conformations of Pen a 1 and analyze the impact of conformation and resolution on the optimal rule-based model.

**Conclusions:**

We find that the optimized rule-based models provide information about the average steric hindrance between binding regions and the probability that antibodies will bind to these regions. These optimized models quantify the variation in aggregate size that results from differences in molecular geometry and from model resolution.

## Background

Computational models are widely used to study biomolecular interactions due to their complexity. Models which are constrained by physicochemical principles are useful because they are based on causality rather than simply correlation, and their parameters, such as molecule copy numbers and binding rates, can be measured independently [1, 2]. Models that enable the incorporation of site-specific details and that can overcome the problem of combinatorial complexity are also highly useful for biomolecular simulations [2–4]. One technique that meets all of the above requirements is rule-based modeling. Rule-based modeling is a technique for studying the site dynamics of biomolecular networks [2, 5], which involves representing biomolecular interactions as local rules. With this method, a set of rules is defined where each rule represents a bidirectional reaction. Each direction of the reaction is assigned a rate law. During simulation, a reaction network is created from which a set of coupled ordinary differential equations (ODEs) is derived. These equations characterize the rates of change of observables (such as chemical species). There are several different formalisms that may be used in creating rule-based models [2, 5]. One disadvantage of the traditional rule-based model is that it does not capture details of molecular geometry. The traditional rule-based model is based on a set of binding rules that only include the number of binding sites on a molecule and do not incorporate geometric information [6, 7]. This limitation of the rule-based modeling approach results in models that are unable to capture the effects of molecular geometry. In this work, our goal is to develop a new, general rule-based model for molecular binding events that implicitly represents molecular geometry by using simple measurements between sections of the molecules to encode steric effects into the rules.

As a model system for our methodology, we look at the process of antigen-antibody aggregation; in particular, we study the binding of IgE-Fc *ε*RI antibody-receptor complexes to the antigen Pen a 1, a common shrimp allergen. An antigen is a substance that is capable of inducing the production of antibodies and binds to them via regions on its surface called epitopes. Studying antigen-antibody aggregation and the structure of the aggregates that form during this process is important for understanding how the allergic response is initiated. The allergic response in humans is set into motion by a tyrosine kinase cascade that results from the crosslinking of IgE-Fc *ε*RI receptor complexes via the binding of the IgE antibodies to an antigen [8]. Antigens may have multiple possible conformations with differing geometric characteristics, which can affect the size and structure of the antigen-antibody aggregates that form. The fold of an allergen is known to play a role in the IgE reactivity of its epitopes [9]. The development of a practical method for aggregate structure prediction based on the geometry of a particular antigen conformation could be useful not only for understanding aggregation, but also for possible manipulation of the antigen geometry to obtain a desired aggregate structure. Various properties of allergens and protein complexes, such as structural stability, flexibility, and dimerization, have been studied using molecular dynamics-based methods [10, 11]; however, such methods would not be useful to study antibody aggregation due to the long timescales involved (on the order of seconds) and the large size and quantity of the aggregates that may form.

In order to obtain aggregate size distribution data on which to base our rule-based models, we employ a previously developed three-dimensional rigid-body Monte Carlo method [12]. This method can explicitly represent molecular geometry and molecular motions. A graph-based structure defines the molecular interactions; ligands and receptors are represented by vertices, and bonds between ligands and receptors are represented by edges. This structure allows for the easy maintenance of aggregation information throughout the simulation, and for the analysis of aggregate structure [12]. Instead of all-atom molecular structures, 3D isosurface representations of the molecules are used, which reduces the simulation to a rigid-body problem and reduces the computational cost [12]. This method can be combined with rule-based modeling in order to quantify the steric effects between allergen binding regions that affect binding site accessibility and to represent the differences in these steric effects caused by variations in molecular curvature. Steric effects in the 3D Monte Carlo model depend not only on molecular geometry but also on the resolution of the 3D molecular model. The resolution of the isosurface models can be reduced through polygon reduction, which has been shown to decrease the number of polygon-to-polygon comparisons that occur during the Monte Carlo simulation [13, 14]; consequently, the computational time of the simulation also reduces. The simulation time has been previously shown to decrease from approximately 20 hours per run for the original isosurface model to 10 hours per run for a model with a polygon reduction of 50 %, and two hours per run for a model with 90 % reduction. However, a reduction in resolution decreases the volume of the molecular models and affects the resulting aggregate size distribution [13, 14].

Our previous work [14] introduced our initial efforts toward using a rule-based method for modeling molecular geometry through capturing the steric effects acting on the various binding regions of the molecule. In this paper, we propose a more general rule-based method to model the geometric effects of molecular binding events. Here, we extend our initial investigation presented in [15] to include the impact of model resolution to the formation of molecular aggregates for all three energy-minimized Pen a 1 conformations studied. This novel rule-based method involves the automation of rule set construction based on a few parameters that encode the steric effects between neighboring binding regions of the antigen. In particular, we apply our method to the modeling of antigen-antibody aggregation and investigate different antigen conformations. We expect that modifying the curvature of the linear Pen a 1 antigen may result in distinct steric effects. We first demonstrate our method using three Pen a 1 molecules with large variations in curvature. Then, we use this method to model the aggregation of IgE-Fc *ε*RI receptor complexes with the shrimp allergen Pen a 1 for three different energy-minimized conformations of the Pen a 1 molecule that have small variations in curvature: native, S-shaped, and U-shaped. We compare the results of this method with the results of the aforementioned Monte Carlo simulations, and we analyze the differences in antigen-antibody aggregate formation for each the conformations. We also perform a resolution study in which we run Monte Carlo simulations for seven resolutions of the 3D model and subsequently use our novel rule-based method to analyze the variation of steric effects and quantify the change in relevant parameters.

### Related work

Our work builds off of computational methods for modeling molecular geometry in biochemical processes, as well as experimental methods to study IgE antibody binding behavior.

#### Rule-based modeling

Biological signaling systems are often comprised of macromolecules that can exist in a large number of functionally distinct states. This number scales exponentially with the amount of modification possibilities [7]. One problem that arises when modeling these systems is the specification problem, i.e. how to specify such a large system.

One solution is implicit specification, which involves the coarse-graining of sets of reactions and parameters into “rules”; the only explicitly specified features in a reaction rule are those which affect the reaction. Rules define the conditions for molecular transformations and interactions, and are associated with rate laws [2]. Some rules define multiple reactions, which means that all of these reactions are associated with the same rate law. The rules can usually be specified independently. Rule-based specification methods include Kappa-calculus [16], BioNetGen [5], ANC [17], and ML-Rules [18]. The Simmune project and the SSC allow the specification of molecules within spatial regions of arbitrary geometries [19].

BioNetGen [5] is a popular rule-based modeling software which uses graph rewriting. The biomolecules are represented by graphs, vertices represent molecular components, and edges represent bonds between these components. Biomolecular complexes are represented by connected sets of graphs. Rules are applied to these graphs and sets of graphs, and the graphs are changed according to the results of the biomolecular interactions specified by these rules [2, 5].

The rule-based methods can be population-based, particle-based, or hybrid. Population-based methods include ODE/PDE numerical integration and the stochastic Gillespie algorithm. In these methods, the application of a rule changes the size of one of the populations, each of which consists of all molecules that share the same state and same species. The system state space can be very large, so methods to reduce it have been introduced [7].

Particle-based rule evaluation involves tracking individual particles (molecules and molecular complexes) through the simulation [2]. This is a network-free method; at any time point, only the existing particles, their states, and the possible reactions for the existing particles are necessary. Spatial particle-based methods include an explicit specification of space, and include SRSim [20] and MCell [21].

Our method differs from traditional approaches to rule-based modeling in that the rules used in our method are constrained by steric effects induced by molecular geometry and 3D model resolution. We introduced a different rule-based method for modeling resolution in [14]; this method used a single set of rules for each molecule conformation with only the rate constants associated with the rules varying with resolution. Additionally, there were four different rate constants that required optimization. On the other hand, the rule-based modeling method presented in this work only requires two parameters that need to be optimized, making it more efficient than our previously developed methodological approach.

#### Geometric molecular modeling

The spatial simulation software SRSim [20] is a rule-based modeling method that allows for the specification of molecule geometry. SRSim integrates rule-based modeling, molecular dynamics, and a stochastic, diffusing-particle simulator. Molecular geometry is provided by the user via data files. Our proposed method is different in that it is a purely rule-based ODE model that does not require any additional data files to run, as the molecular geometry is encoded into the rules themselves. In addition, our method only requires the BioNetGen software to run.

The stochastic, particle-based Meredys software [22] uses Brownian dynamics to simulate reaction-diffusion systems at the mesoscopic level. It requires the specification of details such as molecule positions, molecular geometry, reaction site positions, and reaction types. Our rule-based method is population-based and only requires the distances between pairs of binding regions on a single antigen molecule to create the model.

Computational methods for modeling two-molecule ligand-receptor docking simulate systems on a smaller scale than those studied using our method. Our method uses more realistic geometric molecular models than do existing methods for self-assembly of molecular structures, such as those employing simple bead models [23].

#### Experimental methods

Spatiotemporal analysis using nanoparticles have been developed to understand the process of IgE antibody aggregation. Methods to analyze clustered IgE data include the use of Ripleys and Hopkins statistics [24] as well as hierarchical clustering techniques which attempt to quantify the numbers of sizes of clusters [25]. Spatial data of gold nanoparticle labeled IgE have been imaged on the cell surface using transmission electron microscopy [26]. Tracking quantum dot labeled IgE has provided temporal information including diffusion rates [27]. While these experimental methods can measure attributes about receptor dynamics, neither provide aggregate binding pattern information, making the process of distinguishing linked from simply proximal receptors challenging.

## Methods

In this section, we present a rule-based model of the shrimp allergen Pen a 1 that encodes the steric hindrances between the IgE binding regions of the allergen within the set of rules. We first briefly discuss the binding sites and binding regions of the Pen a 1 molecule. Next, we outline the assumptions that were made in the development of this rule-based model. We then describe our methods for determining the steric hindrances between the binding regions, constructing a set of rules based on these steric effects, optimizing the rate constants, and implementing the rule-based simulation. We explain how the probabilities of formation of various aggregate sizes were calculated. Lastly, we describe the Monte Carlo rigid-body model and how we compared the results of our rule-based model to that of the Monte Carlo simulation.

### Pen a 1 structure and valency

The Pen a 1 allergen possesses a double-stranded coiled structure. All-atom structures of shrimp tropomyosin were obtained from the Protein Data Bank (PDB:1CG1) and the Structural Database of Allergenic Proteins (SDAP Model #284) [28, 29]. The Pen a 1 model used contains 568 amino acids and 4,577 atoms. Experimental studies have predicted that Pen a 1 has 16-18 binding sites, which can be grouped into five general binding regions per strand [28, 30, 31]. The amino acid sequences of the binding sites are listed in [28]. The all-atom structure of the IgE-Fc *ε*RI receptor complex was obtained from [32]. It contains 1,709 amino acids and 13,477 atoms.

**Fig. 1:**
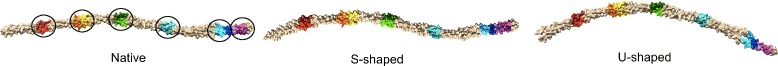
Three conformations of the all-atom molecular structure of the shrimp tropomyosin Pen a 1 (tan). The IgE binding regions are circled. The IgE binding regions (various colors) are located in five regions per strand, although for our rule-based model, we have split the longer rightmost region into two separate regions so that there are six binding regions per strand. We use the native configuration (*left*), an accentuated curve version called S-Shaped (*middle*), and a U-Shaped configuration (*right*). In this paper, we label the regions on the native configuration (from left to right) as regions A, B, C, D, E, and F

We generate 3 energetically feasible configurations of the Pen a 1 antigen (Fig. 1). We split the longest region into two binding regions (E and F) in our rule-based model such that Pen a 1 has six binding regions per strand, with 12 total regions in our model (Fig. 1, Native). This is because the longest region is significantly larger than the other regions and contains 8 epitopes. Due to the symmetric structure of Pen a 1, we allow distinct IgE molecules to bind to the same region on opposite strands simultaneously.

### Rule-based model assumptions

**Fig. 2:**
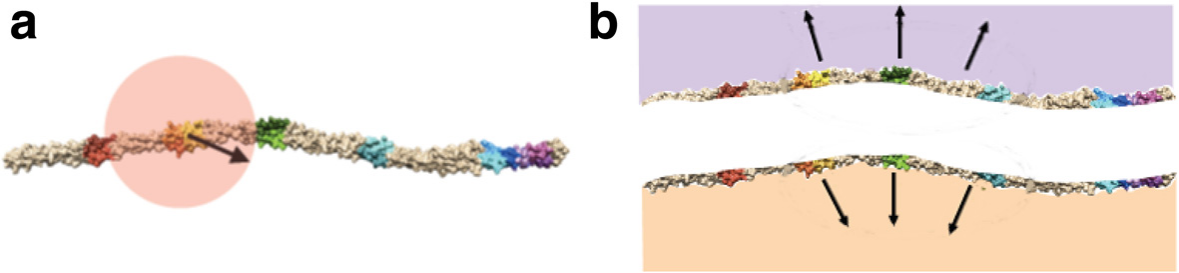
Rule-based modeling with steric effects. **a** Circles represent a possible region of steric hindrance around the yellow/orange binding region where the radius of the circle represents the cutoff distance *d*_*c*_. **b** Types of curvature on the Pen a 1 molecule, visualized split into two strands. Accessibility to the regions may be affected when neighboring regions are occupied depending on either positive curvature (*top*), or negative curvature (*bottom*)

In order to simplify our rule-based model and to ensure that the number of rules in the rule set does not become too large for implementation, we make several practical assumptions when constructing our rule sets. Firstly, we assume that an IgE can only bind to a single binding region on the Pen a 1 molecule, and that an IgE cannot be bound to multiple regions simultaneously. For simplicity, and since the Monte Carlo data does not incorporate crosslinking, our rules forbid crosslinking Pen a 1 molecules through IgE binding. As discussed previously, we simplify our model further by assuming that there are only 12 total binding regions on the Pen a 1 molecule (six per strand), and that each IgE can only bind to one of these 12 regions. Finally, we assume that each of the two strands in the Pen a 1 molecule binds independently of the other strand. An IgE bound to a region on one strand does not, in any way, affect the probability of an IgE receptor binding to a region on the opposite strand. Figure 2 shows Pen a 1 divided into strands.

### Determining steric effects and rules for various Pen a 1 conformations and model resolutions

The *cutoff distanced*_*c*_ is an important parameter in this study that is used to help us automate rule set construction. In this paper, we use the term *cutoff distance* to specify the maximum distance separating two binding regions on a strand of Pen a 1 at which the two regions have steric effects on each other (Fig. 2a), meaning that if one of these regions is bound to a receptor, then the probability that the other region can be bound to a receptor is reduced. The cutoff distance determines the rule set of the rule-based model. For each conformation and model resolution, the cutoff distance is varied and tested to find its optimal value, which is the value that results in a rule-based model that most accurately represents the aggregate size probability data obtained from the Monte Carlo simulation.

The Pen a 1 molecule is flexible and has various possible conformations due to local energy minima. In our model, IgE-Fc *ε*RI receptor complexes are bound to a two-dimensional cell membrane, and the Pen a 1 molecule is constrained to move on a 2D surface. Each Pen a 1 conformation possesses different curvature properties. In this study, we focus on native [13, 14], S-shaped, and U-shaped conformations (Fig. 1). The conformations for our study were designed using standalone Foldit [33] and were energy-minimized using MOIL software [34].

The molecular curvature around a binding region, along with the IgE receptors bound to neighboring binding regions, may cause steric hindrance around the binding region, i.e., IgE receptors may be prevented from binding to the region due to the region being blocked by receptors bound to neighboring regions. A neighboring region is any region that is close enough to the region under consideration to potentially block it if bound to a receptor. There are three general categories of steric effects that we consider in our model, which are illustrated in Fig. 2b. Firstly, if all neighboring regions are unbound, there is no steric hindrance imposed on a binding region. Secondly, if the molecular curvature around a binding region is positive (Fig. 2b, *top*), receptors bound to neighboring regions are unlikely to affect the accessibility to this region since positive curvature around two regions pulls them farther apart (the linear distance between the regions increases). Lastly, if the molecular curvature around a binding region is negative (Fig. 2b, *bottom*), receptors bound to neighboring regions may reduce the accessibility to this region since negative curvature around two regions brings them closer together (the linear distance between the regions decreases).

The distances between each pair of binding regions on each strand of the Pen a 1 molecule were measured for each Pen a 1 conformation studied. For region pairs located in an area of negative curvature, the linear distance between the regions was measured. Otherwise, the distance of a free-form path along the molecule between the two regions was used. This difference in distance measurement accounts for the variation in steric effects that results from different types of curvature. If the distance between a pair of regions is less than the specified cutoff distance, then those two regions are considered to exert steric effects on each other, and the steric hindrance is encoded into the binding rules for those regions.

For this study, the first rule construction method we tested used a simple set of rules in which every rule has the same rate constant *k*_*f*1_, and no binding is allowed onto a region that has any steric effects exerted on it by any other region. The second method builds on the first method by allowing binding onto these regions with a reduced, but non-zero, probability set by a separate forward rate constant *k*_*f*2_ assigned to rules that specify a steric hindrance between regions.

Given that *r* is the distance between two binding regions and *d*_*c*_ is the cutoff distance, if one of these two binding regions is occupied and the other region is free, the binding rate constant *k*_*f*_ for a receptor binding to the free region is assigned according to the following:

$$\begin{array}{@{}rcl@{}} \text{If}~r > d_{c}, &\text{then }& k_{f} = k_{f1} = 1.0~\text{molecule}^{-1}\mathrm{s}^{-1}, \\ \text{If}~r \le d_{c}, &\text{then }& k_{f} = k_{f2} < k_{f1}. \end{array} $$

The rate constant *k*_*f*2_ was determined by performing an optimization and fitting the resulting aggregate size data to that of the 3D Monte Carlo model.

### Rate constant optimization

Because the cutoff distance is unknown, this parameter was varied from 3.0 nm to 20.0 nm in 0.1 nm increments, with the rule set being reconstructed for each cutoff distance. An optimization of *k*_*f*2_ was performed for every cutoff distance.

The forward rate constant *k*_*f*2_ for each Pen a 1 conformation and model resolution was optimized using an adaptive algorithm based on the Metropolis-Hastings algorithm. This algorithm finds a minimum of the residual sum-of-squares (*σ*) between the RBM data and the Monte Carlo data. If the *σ* value for a new rate constant $k_{f2_{n}}$ is *σ*_*n*_, the current *σ* value is *σ*_*c*_, and the current rate constant is *k*_*f*2_, then the rate constant is determined according to the following:

$$\begin{aligned} &\text{If} ~~~ \sigma_{n} > \sigma_{c},~ \text{then}~ \sigma_{c} = \sigma_{n}~ \textsl{\&}~ k_{f2} = k_{f2_{n}}~\text{w/~prob.}~e^{-\Delta{\sigma}/T}, \\ &\text{If}~~~ \sigma_{n} > \sigma_{c}, ~\text{then}~ \sigma_{c} = \sigma_{c} ~\textsl{\&}~ k_{f2} = k_{f2}~\text{w/~prob.}~1-e^{-\Delta{\sigma}/T}, \\ &\text{If} ~~~ \sigma_{n} \le \sigma_{c}, ~\text{then}~ \sigma_{c} = \sigma_{n}~\textsl{\&}~ k_{f2} = k_{f2_{n}}~\text{w/ prob.}~1. \end{aligned} $$

If *σ*_*n*_ is higher than *σ*_*c*_, then *σ*_*n*_ (and $k_{f2_{n}}$) are accepted with a probability dependent on the difference between *σ*_*n*_ and *σ*_*c*_, *Δσ*, and the simulated annealing temperature *T*. If the new value is accepted, then the rate constant is incremented according to the specified step size, and the new rate constant is tested. Adaptive rate constant step sizes of 1 ×10^−4^ molecule ^−1^*s*^−1^ and 1 ×10^−5^ molecule ^−1^*s*^−1^ were used (if *σ* is decreasing, the smaller step size is used to find and test a new rate constant; otherwise, the larger step size is used). However, if the new value is rejected, then the algorithm will choose a new rate constant at random from over the entire allowed range. The algorithm was allowed to search over the range 0.00 to 0.40 molecule ^−1^*s*^−1^. We empirically determined from previous scans of *k*_*f*2_ that *σ* for any value of *k*_*f*2_ greater than 0.40 molecule ^−1^*s*^−1^ would be too high to be acceptable.

### Rule-based model implementation

The rule-based model is specified using the BioNetGen language [5] and implemented with the RuleBender program [35]. RuleBender generates the ODEs associated with the binding rules and tracks the aggregates that are formed as the IgE receptors bind to the Pen a 1 molecules in an ODE simulation. Each strand of the two-stranded molecules is simulated separately. Each run takes no more than 10 seconds to reach the steady state.

Another version of the rule set with forward binding rate constants proportional to the number of binding sites in the region was also tested, but did not yield aggregate size data that fit better to the Monte Carlo data than the rule sets used.

### Aggregate size probability calculation

Our model assumes that Pen a 1 is comprised of two strands, which we refer to here as strand *I* and strand *II*. Therefore, the probability of formation of an aggregate of a certain size is calculated by combining the independent probabilities of formation of each strand. The probability *P*(*n*) to form an aggregate of size *n* is given by:

1 $$ P(n \le 6) = \sum\limits_{m=0}^{n}P_{I}(m)P_{II}(n-m),  $$

2 $$ P(n > 6) = \sum\limits_{m=n-6}^{6}P_{I}(m)P_{II}(n-m),  $$

where *P*_*I*(*II*)_(*n*) is the independent probability of forming an aggregate of size *n* in strand *I*(*II*).

### Variation of rule set with curvature

**Fig. 3:**
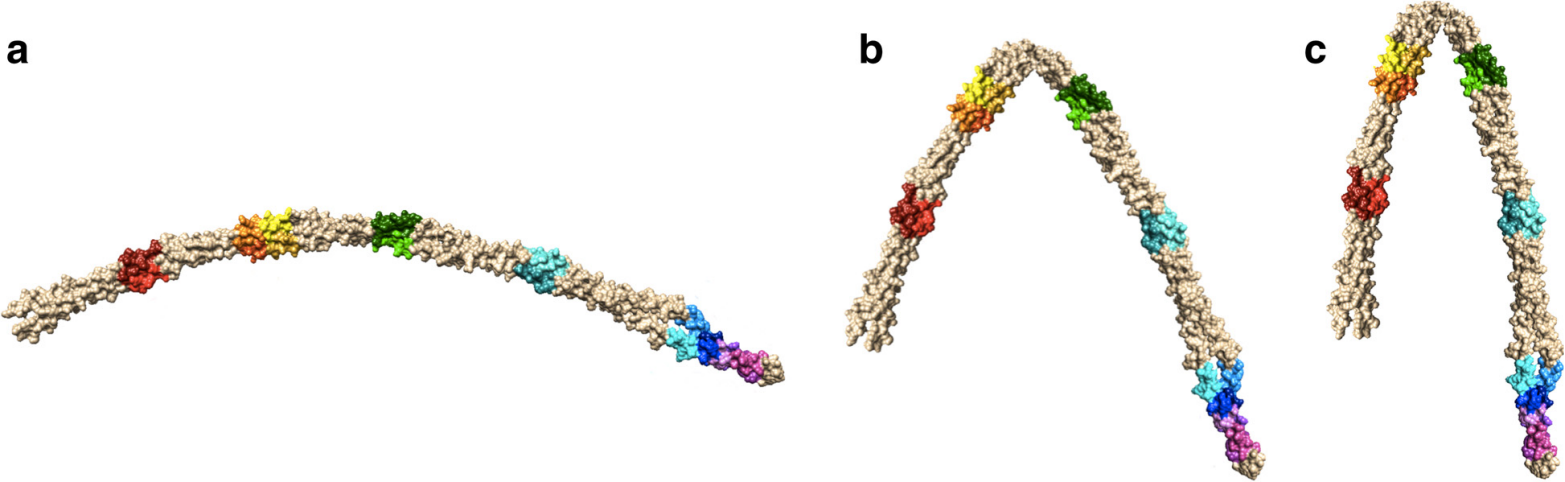
Visualizations of the **a** U-shaped, **b** 45-degree-rotated, and **c** 60-degree-rotated molecular structures. (It should be noted that the latter two molecules are not energy-minimized conformations and are only presented here for the purpose of demonstrating our rule construction method)

In order to more clearly illustrate how the set of binding rules for a given molecule is affected by molecular curvature using our method of rule construction, we present an example of a U-shaped Pen a 1 molecule with varying curvature. We look at three molecules: the U-shaped molecule seen in Fig. 1, the same molecule with its two ends rotated inward by 45 degrees, and the same molecule with its two ends rotated inward by 60 degrees (see Fig. 3). It should be noted that the latter two molecules are not energy-minimized conformations and are only presented here for the purpose of demonstrating our geometric rule construction method. The rule sets for strand I are shown for the 45-degree-rotated molecular structure (Table 1), the 60-degree-rotated molecular structure (Table 2), and the U-shaped Pen a 1 molecule (Table 3).

**Table 1:** Rule set for Strand I (*T*_*I*_) of the 45-degree-rotated molecular structure. Letters in parentheses are binding sites. Omitted letters are free or occupied. The IgE subscript shows which site it is bound to

Binding site	Reaction rule	Binding rate
A	*T*_*I*_(A,B,C) + IgE →*T*_*I*_(IgE _*A*_,B,C)	k _*f*1_
	*T*_*I*_(A,IgE _*B*_,C) + IgE →*T*_*I*_(IgE _*A*_,IgE _*B*_,C)	k _*f*2_
	*T*_*I*_(A,B,IgE _*C*_) + IgE →*T*_*I*_(IgE _*A*_,B,IgE _*C*_)	k _*f*2_
	*T*_*I*_(A,IgE _*B*_,IgE _*C*_) + IgE →*T*_*I*_(IgE _*A*_,IgE _*B*_,IgE _*C*_)	k _*f*2_
B	*T*_*I*_(A,B,C) + IgE →*T*_*I*_(A,IgE _*B*_,C)	k _*f*1_
	*T*_*I*_(IgE _*A*_,B,C) + IgE →*T*_*I*_(IgE _*A*_,IgE _*B*_,C)	k _*f*2_
	*T*_*I*_(A,B,IgE _*C*_) + IgE →*T*_*I*_(A,IgE _*B*_,IgE _*C*_)	k _*f*2_
	*T*_*I*_(IgE _*A*_,B,IgE _*C*_) + IgE →*T*_*I*_(IgE _*A*_,IgE _*B*_,IgE _*C*_)	k _*f*2_
C	*T*_*I*_(A,B,C,D) + IgE →*T*_*I*_(A,B,IgE _*C*_,D)	k _*f*1_
	*T*_*I*_(IgE _*A*_,B,C,D) + IgE →*T*_*I*_(IgE _*A*_,B,IgE _*C*_,D)	k _*f*2_
	*T*_*I*_(A,IgE _*B*_,C,D) + IgE →*T*_*I*_(A,IgE _*B*_,IgE _*C*_,D)	k _*f*2_
	*T*_*I*_(A,B,C,IgE _*D*_) + IgE →*T*_*I*_(A,B,IgE _*C*_,IgE _*D*_)	k _*f*2_
	*T*_*I*_(IgE _*A*_,IgE _*B*_,C,D) + IgE →*T*_*I*_(IgE _*A*_,IgE _*B*_,IgE _*C*_,D)	k _*f*2_
	*T*_*I*_(IgE _*A*_,B,C,IgE _*D*_) + IgE →*T*_*I*_(IgE _*A*_,B,IgE _*C*_,IgE _*D*_)	k _*f*2_
	*T*_*I*_(A,IgE _*B*_,C,IgE _*D*_) + IgE →*T*_*I*_(A,IgE _*B*_,IgE _*C*_,IgE _*D*_)	k _*f*2_
	*T*_*I*_(IgE _*A*_,IgE _*B*_,C,IgE _*D*_) + IgE →*T*_*I*_(IgE _*A*_,IgE _*B*_,IgE _*C*_,IgE _*D*_)	k _*f*2_
D	*T*_*I*_(C,D,E) + IgE →*T*_*I*_(C,IgE _*D*_,E)	k _*f*1_
	*T*_*I*_(IgE _*C*_,D,E) + IgE →*T*_*I*_(IgE _*C*_,IgE _*D*_,E)	k _*f*2_
	*T*_*I*_(C,D,IgE _*E*_) + IgE →*T*_*I*_(C,IgE _*D*_,IgE _*E*_)	k _*f*2_
	*T*_*I*_(IgE _*C*_,D,IgE _*E*_) + IgE →*T*_*I*_(IgE _*C*_,IgE _*D*_,IgE _*E*_)	k _*f*2_
E	*T*_*I*_(D,E,F) + IgE →*T*_*I*_(D,IgE _*E*_,F)	k _*f*1_
	*T*_*I*_(IgE _*D*_,E,F) + IgE →*T*_*I*_(IgE _*D*_,IgE _*E*_,F)	k _*f*2_
	*T*_*I*_(D,E,IgE _*F*_) + IgE →*T*_*I*_(D,IgE _*E*_,IgE _*F*_)	k _*f*2_
	*T*_*I*_(IgE _*D*_,E,IgE _*F*_) + IgE →*T*_*I*_(IgE _*D*_,IgE _*E*_,IgE _*F*_)	k _*f*2_
F	*T*_*I*_(E,F) + IgE →*T*_*I*_(E,IgE _*F*_)	k _*f*1_
	*T*_*I*_(IgE _*E*_,F) + IgE →*T*_*I*_(IgE _*E*_,IgE _*F*_)	k _*f*2_

**Table 2:** Rule set for Strand I (*T*_*I*_) of the 60-degree-rotated molecular structure. Letters in parentheses are binding sites. Omitted letters are free or occupied. The IgE subscript shows which site it is bound to

Binding site	Reaction rule	Binding rate
A	*T*_*I*_(A,B,C,D) + IgE →*T*_*I*_(IgE _*A*_,B,C,D)	k _*f*1_
	*T*_*I*_(A,IgE _*B*_,C,D) + IgE →*T*_*I*_(IgE _*A*_,IgE _*B*_,C,D)	k _*f*2_
	*T*_*I*_(A,B,IgE _*C*_,D) + IgE →*T*_*I*_(IgE _*A*_,B,IgE _*C*_,D)	k _*f*2_
	*T*_*I*_(A,B,C,IgE _*D*_) + IgE →*T*_*I*_(IgE _*A*_,B,C,IgE _*D*_)	k _*f*2_
	*T*_*I*_(A,IgE _*B*_,IgE _*C*_,D) + IgE →*T*_*I*_(IgE _*A*_,IgE _*B*_,IgE _*C*_,D)	k _*f*2_
	*T*_*I*_(A,IgE _*B*_,C,IgE _*D*_) + IgE →*T*_*I*_(IgE _*A*_,IgE _*B*_,C,IgE _*D*_)	k _*f*2_
	*T*_*I*_(A,B,IgE _*C*_,IgE _*D*_) + IgE →*T*_*I*_(IgE _*A*_,B,IgE _*C*_,IgE _*D*_)	k _*f*2_
	*T*_*I*_(A,IgE _*B*_,IgE _*C*_,IgE _*D*_) + IgE →*T*_*I*_(IgE _*A*_,IgE _*B*_,IgE _*C*_,IgE _*D*_)	k _*f*2_
B	*T*_*I*_(A,B,C) + IgE →*T*_*I*_(A,IgE _*B*_,C)	k _*f*1_
	*T*_*I*_(IgE _*A*_,B,C) + IgE →*T*_*I*_(IgE _*A*_,IgE _*B*_,C)	k _*f*2_
	*T*_*I*_(A,B,IgE _*C*_) + IgE →*T*_*I*_(A,IgE _*B*_,IgE _*C*_)	k _*f*2_
	*T*_*I*_(IgE _*A*_,B,IgE _*C*_) + IgE →*T*_*I*_(IgE _*A*_,IgE _*B*_,IgE _*C*_)	k _*f*2_
C	*T*_*I*_(A,B,C,D) + IgE →*T*_*I*_(A,B,IgE _*C*_,D)	k _*f*1_
	*T*_*I*_(IgE _*A*_,B,C,D) + IgE →*T*_*I*_(IgE _*A*_,B,IgE _*C*_,D)	k _*f*2_
	*T*_*I*_(A,IgE _*B*_,C,D) + IgE →*T*_*I*_(A,IgE _*B*_,IgE _*C*_,D)	k _*f*2_
	*T*_*I*_(A,B,C,IgE _*D*_) + IgE →*T*_*I*_(A,B,IgE _*C*_,IgE _*D*_)	k _*f*2_
	*T*_*I*_(IgE _*A*_,IgE _*B*_,C,D) + IgE →*T*_*I*_(IgE _*A*_,IgE _*B*_,IgE _*C*_,D)	k _*f*2_
	*T*_*I*_(IgE _*A*_,B,C,IgE _*D*_) + IgE →*T*_*I*_(IgE _*A*_,B,IgE _*C*_,IgE _*D*_)	k _*f*2_
	*T*_*I*_(A,IgE _*B*_,C,IgE _*D*_) + IgE →*T*_*I*_(A,IgE _*B*_,IgE _*C*_,IgE _*D*_)	k _*f*2_
	*T*_*I*_(IgE _*A*_,IgE _*B*_,C,IgE _*D*_) + IgE →*T*_*I*_(IgE _*A*_,IgE _*B*_,IgE _*C*_,IgE _*D*_)	k _*f*2_
D	*T*_*I*_(A,B,C,D,E) + IgE →*T*_*I*_(A,B,C,IgE _*D*_,E)	k _*f*1_
	*T*_*I*_(IgE _*A*_,B,C,D,E) + IgE →*T*_*I*_(IgE _*A*_,B,C,IgE _*D*_,E)	k _*f*2_
	*T*_*I*_(A,IgE _*B*_,C,D,E) + IgE →*T*_*I*_(A,IgE _*B*_,C,IgE _*D*_,E)	k _*f*2_
	*T*_*I*_(A,B,IgE _*C*_,D,E) + IgE →*T*_*I*_(A,B,IgE _*C*_,IgE _*D*_,E)	k _*f*2_
	*T*_*I*_(A,B,C,D,IgE _*E*_) + IgE →*T*_*I*_(A,B,C,IgE _*D*_,IgE _*E*_)	k _*f*2_
	*T*_*I*_(IgE _*A*_,IgE _*B*_,C,D,E) + IgE →*T*_*I*_(IgE _*A*_,IgE _*B*_,C,IgE _*D*_,E)	k _*f*2_
	*T*_*I*_(IgE _*A*_,B,IgE _*C*_,D,E) + IgE →*T*_*I*_(IgE _*A*_,B,IgE _*C*_,IgE _*D*_,E)	k _*f*2_
	*T*_*I*_(IgE _*A*_,B,C,D,IgE _*E*_) + IgE →*T*_*I*_(IgE _*A*_,B,C,IgE _*D*_,IgE _*E*_)	k _*f*2_
	*T*_*I*_(A,IgE _*B*_,IgE _*C*_,D,E) + IgE →*T*_*I*_(A,IgE _*B*_,IgE _*C*_,IgE _*D*_,E)	k _*f*2_
	*T*_*I*_(A,IgE _*B*_,C,D,IgE _*E*_) + IgE →*T*_*I*_(A,IgE _*B*_,C,IgE _*D*_,IgE _*E*_)	k _*f*2_
	*T*_*I*_(A,B,IgE _*C*_,D,IgE _*E*_) + IgE →*T*_*I*_(A,B,IgE _*C*_,IgE _*D*_,IgE _*E*_)	k _*f*2_
	*T*_*I*_(IgE _*A*_,IgE _*B*_,IgE _*C*_,D,E) + IgE →*T*_*I*_(IgE _*A*_,IgE _*B*_,IgE _*C*_,IgE _*D*_,E)	k _*f*2_
	*T*_*I*_(IgE _*A*_,IgE _*B*_,C,D,IgE _*E*_) + IgE →*T*_*I*_(IgE _*A*_,IgE _*B*_,C,IgE _*D*_,IgE _*E*_)	k _*f*2_
	*T*_*I*_(IgE _*A*_,B,IgE _*C*_,D,IgE _*E*_) + IgE →*T*_*I*_(IgE _*A*_,B,IgE _*C*_,IgE _*D*_,IgE _*E*_)	k _*f*2_
	*T*_*I*_(A,IgE _*B*_,IgE _*C*_,D,IgE _*E*_) + IgE →*T*_*I*_(A,IgE _*B*_,IgE _*C*_,IgE _*D*_,IgE _*E*_)	k _*f*2_
	*T*_*I*_(IgE _*A*_,IgE _*B*_,IgE _*C*_,D,IgE _*E*_) + IgE →	k _*f*2_
	*T*_*I*_(IgE _*A*_,IgE _*B*_,IgE _*C*_,IgE _*D*_,IgE _*E*_)	
E	*T*_*I*_(D,E,F) + IgE →*T*_*I*_(D,IgE _*E*_,F)	k _*f*1_
	*T*_*I*_(IgE _*D*_,E,F) + IgE →*T*_*I*_(IgE _*D*_,IgE _*E*_,F)	k _*f*2_
	*T*_*I*_(D,E,IgE _*F*_) + IgE →*T*_*I*_(D,IgE _*E*_,IgE _*F*_)	k _*f*2_
	*T*_*I*_(IgE _*D*_,E,IgE _*F*_) + IgE →*T*_*I*_(IgE _*D*_,IgE _*E*_,IgE _*F*_)	k _*f*2_
F	*T*_*I*_(E,F) + IgE →*T*_*I*_(E,IgE _*F*_)	k _*f*1_
	*T*_*I*_(IgE _*E*_,F) + IgE →*T*_*I*_(IgE _*E*_,IgE _*F*_)	k _*f*2_

**Table 3:** Rule set for Strand I (*T*_*I*_) of 0 % reduced (full isosurface) model of Pen a 1 for all energy-minimized conformations (native, S-shaped, U-shaped). Letters in parentheses are binding sites. Omitted letters are free or occupied. The IgE subscript shows which site it is bound to

Binding site	Reaction rule	Binding rate
A	*T*_*I*_(A,B) + IgE →*T*_*I*_(IgE _*A*_,B)	k _*f*1_
	*T*_*I*_(A,IgE _*B*_) + IgE →*T*_*I*_(IgE _*A*_,IgE _*B*_)	k _*f*2_
B	*T*_*I*_(A,B,C) + IgE →*T*_*I*_(A,IgE _*B*_,C)	k _*f*1_
	*T*_*I*_(IgE _*A*_,B,C) + IgE →*T*_*I*_(IgE _*A*_,IgE _*B*_,C)	k _*f*2_
	*T*_*I*_(A,B,IgE _*C*_) + IgE →*T*_*I*_(A,IgE _*B*_,IgE _*C*_)	k _*f*2_
	*T*_*I*_(IgE _*A*_,B,IgE _*C*_) + IgE →*T*_*I*_(IgE _*A*_,IgE _*B*_,IgE _*C*_)	k _*f*2_
C	*T*_*I*_(B,C,D) + IgE →*T*_*I*_(B,IgE _*C*_,D)	k _*f*1_
	*T*_*I*_(IgE _*B*_,C,D) + IgE →*T*_*I*_(IgE _*B*_,IgE _*C*_,D)	k _*f*2_
	*T*_*I*_(B,C,IgE _*D*_) + IgE →*T*_*I*_(B,IgE _*C*_,IgE _*D*_)	k _*f*2_
	*T*_*I*_(IgE _*B*_,C,IgE _*D*_) + IgE →*T*_*I*_(IgE _*B*_,IgE _*C*_,IgE _*D*_)	k _*f*2_
D	*T*_*I*_(C,D,E) + IgE →*T*_*I*_(C,IgE _*D*_,E)	k _*f*1_
	*T*_*I*_(IgE _*C*_,D,E) + IgE →*T*_*I*_(IgE _*C*_,IgE _*D*_,E)	k _*f*2_
	*T*_*I*_(C,D,IgE _*E*_) + IgE →*T*_*I*_(C,IgE _*D*_,IgE _*E*_)	k _*f*2_
	*T*_*I*_(IgE _*C*_,D,IgE _*E*_) + IgE →*T*_*I*_(IgE _*C*_,IgE _*D*_,IgE _*E*_)	k _*f*2_
E	*T*_*I*_(D,E,F) + IgE →*T*_*I*_(D,IgE _*E*_,F)	k _*f*1_
	*T*_*I*_(IgE _*D*_,E,F) + IgE →*T*_*I*_(IgE _*D*_,IgE _*E*_,F)	k _*f*2_
	*T*_*I*_(D,E,IgE _*F*_) + IgE →*T*_*I*_(D,IgE _*E*_,IgE _*F*_)	k _*f*2_
	*T*_*I*_(IgE _*D*_,E,IgE _*F*_) + IgE →*T*_*I*_(IgE _*D*_,IgE _*E*_,IgE _*F*_)	k _*f*2_
F	*T*_*I*_(E,F) + IgE →*T*_*I*_(E,IgE _*F*_)	k _*f*1_
	*T*_*I*_(IgE _*E*_,F) + IgE →*T*_*I*_(IgE _*E*_,IgE _*F*_)	k _*f*2_

**Fig. 4:**
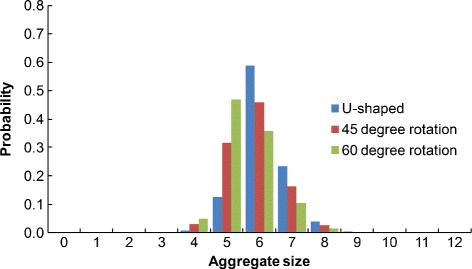
Comparison of rule-based model aggregate size distributions for three curvatures. Results for the U-shaped, 45-degree-rotated, and 60-degree-rotated molecular structures are shown

For each of these three molecules, the distances between each pair of binding regions were measured, and the rule sets for each molecule were constructed according to these distances. For the purpose of comparing how molecular curvature affects the rule set, the cutoff distance was fixed at 8.1 nm, and the forward rate constant *k*_*f*2_ was fixed at 0.005 molecule ^−1^*s*^−1^. The rule-based model for each molecule was simulated and the antibody aggregate size probabilities were calculated (see Fig. 4). We observe that as the degree of curvature of the molecule increases, the aggregate size distribution shifts towards smaller aggregate sizes, which corresponds to the increase in steric effects between binding regions encoded in the rule sets.

### Monte Carlo geometric simulation

We compared the aggregate size distributions from our rule-based model to those of a previously developed Monte Carlo model [12] that uses 3-D rigid body models of the antigens and receptors. Initially, the molecules are randomly positioned within the bounding volume with no bonds present. Then, during the simulation, at every time step, the positions of the molecules change according to a combination of random sampling and biological constraints such as molecular speeds, binding rates, and unbinding rates. Also, at every time step, any two binding sites (on two separate molecules) within binding distance of each other will bind with a probability determined by the binding rate. The stability of the number of edges in the graph-based structure can be used to determine when to stop the simulation. Since the simulation models activity taking place on the surface of a cell membrane, the molecules can translate on the XY plane and rotate about the Z axis. Aggregate size distributions are collected from packed structures after the simulation reaches a steady state.

It should be noted that experimental data pertaining to the aggregation of IgE-Fc *ε*RI receptor complexes onto the Pen a 1 molecule is not readily available as of the date this work was written. Therefore, we cannot compare the computational results of this work to experiment. In addition, the 3D Monte Carlo simulation does not include energetics, which limits our understanding of receptor binding to Pen a 1 and is the reason why we cannot derive the forward rate constants directly from the simulation.

**Fig. 5:**
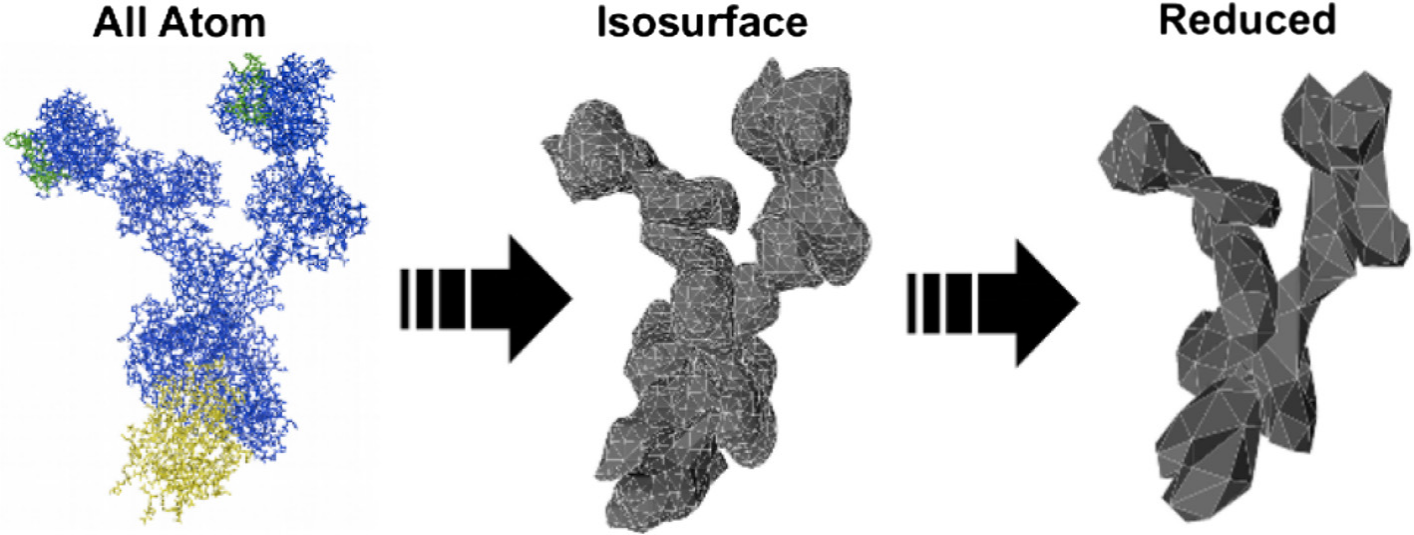
Creation of 3D molecular models at various resolutions. Isosurface models and subsequent polygon reduction are used to generate these models

The 3D molecular models of the Monte Carlo simulation were created by generating isosurface models of the all-atom molecular structures using UCSF Chimera [36]. Since single Monte Carlo runs can require over 20 hours of computation [14], it would be preferable to use lower resolution models to reduce computation time. However, lower resolution models can also introduce simulation effects that are difficult to quantify. We created 3D models with different resolutions and used our novel rule-based method to examine how steric effects vary with resolution. In order to create 3D models with a lower resolution, we performed polygon reduction using Autodesk Maya [37] to reduce the number of polygons by 25 %, 50 %, 65 %, 75 %, 90 %, and 95 % (see Fig. 5 for an illustration). In our study, we also used the original isosurface models, which we refer to as having 0 % polygon reduction.

### Calculating differences from Monte Carlo geometric simulation and Rule-based model

In order to quantify the difference between the Monte Carlo and rule-based modeling aggregate sizes for each conformation and model resolution, the residual sum-of-squares (*σ*) normalized by the total number of possible aggregate sizes (*N*=13) was calculated for each conformation and model resolution. The equation used to calculate the normalized *σ* is:

3 $$ \sigma = \frac{\sum_{i=1}^{N}\left(P_{MC}^{i} - P_{RBM}^{i}\right)^{2}}{N},  $$

where *N* is the total number of possible aggregate sizes in a histogram (each histogram has the same number of possible aggregate sizes), $P_{MC}^{i}$ is the occurrence probability of the *i*^*th*^ aggregate size of the Monte Carlo data, and $P_{RBM}^{i}$ is the occurrence probability of the *i*^*th*^ aggregate size of the rule-based modeling data.

Since the data points used in this calculation are probabilities, the maximum possible normalized *σ* is 1, and the minimum possible normalized *σ* (corresponding to two identical histograms) is zero.

## Results and Discussion

### Experimental setup

#### Monte Carlo simulation

The environment of the Monte Carlo simulations was a 200 nm x 200 nm (40,000 nm^2^) discrete membrane with non-periodic boundaries. For each run, one Pen a 1 molecule and 24 IgE-Fc *ε*RI receptor complexes were simulated, such that the receptor density was ∼600 receptors/ *μ*m^2^. Sixty runs were performed for each Pen a 1 conformation. The values of binding and unbinding rates for Pen a 1 are currently unknown, thus we cannot determine kinetics from our simulation, only possible packing structures. In our simulations we use binding and unbinding rates that were determined for a synthetic IgE antigen [38] with values 1.0 molecule ^−1^*s*^−1^ and 0.01 s ^−1^ respectively. The diffusion coefficient 0.09 *μ*m^2^/s of IgE-Fc *ε*RI found in [27] was used for all molecules. A time step of 10 *μ*s was used, and every experiment was run for 500,000 time steps, which is long enough for the simulations to reach a steady state.

The reduction in speed of aggregates as they increase in size [27], which has not been fully quantified, is included in the simulation by reducing the diffusion coefficient of an aggregate such that it is inversely proportional to the size of the aggregate. For example, the diffusion coefficient of an aggregate containing five receptors would be 1/5 of the original diffusion coefficient. The method of speed reduction implemented only affects aggregation kinetics and does not have a significant effect on the packing structure of aggregates at equilibrium.

The Monte Carlo simulation code was developed using the Parasol Motion Planning Library (PMPL). The simulations were run on a supercomputer housed at the University of New Mexico, utilizing single cores of Intel Xeon E5645 processors with 4 GB of RAM per processor.

#### Rule-based modeling

The rule-based model was specified in the BioNetGen language, and ODE simulations were conducted on these models using RuleBender [35]. In each experiment, 100 Pen a 1 antigen molecules and 1000 receptors were simulated. Because each of the two strands was simulated individually, the total population included 100 strand I molecules, 100 strand II molecules, and 1000 receptors. Each experiment was run for 1000 time steps, long enough for the simulation to reach a steady state, using a time step of 0.01 s. Three Pen a 1 conformations were used (see Fig. 1), which are made distinct in our simulations only by the distance between binding regions.

### Rule sets

The optimized cutoff distance corresponds to an optimal set of binding rules for each conformation of Pen a 1 at 0 % polygon reduction.

The optimal rule sets were found to be the same for all three conformations, although the optimized rate constant *k*_*f*2_ varies based on conformation. These rule sets are shown for strand I (Table 3) and strand II (Table 4) of the Pen a 1 molecule.

**Table 4:** Rule set for Strand II (*T*_*II*_) of 0 % reduced (full isosurface) model of Pen a 1 for all energy-minimized conformations (native, S-shaped, U-shaped). Letters in parentheses are binding sites. Omitted letters are free or occupied. The IgE subscript shows which site it is bound to

Binding site	Reaction rule	Binding rate
A	*T*_*II*_(A,B) + IgE →*T*_*II*_(IgE _*A*_,B)	k _*f*1_
	*T*_*II*_(A,IgE _*B*_) + IgE →*T*_*II*_(IgE _*A*_,IgE _*B*_)	k _*f*2_
B	*T*_*II*_(A,B,C) + IgE →*T*_*II*_(A,IgE _*B*_,C)	k _*f*1_
	*T*_*II*_(IgE _*A*_,B,C) + IgE →*T*_*II*_(IgE _*A*_,IgE _*B*_,C)	k _*f*2_
	*T*_*II*_(A,B,IgE _*C*_) + IgE →*T*_*II*_(A,IgE _*B*_,IgE _*C*_)	k _*f*2_
	*T*_*II*_(IgE _*A*_,B,IgE _*C*_) + IgE →*T*_*II*_(IgE _*A*_,IgE _*B*_,IgE _*C*_)	k _*f*2_
C	*T*_*II*_(B,C,D) + IgE →*T*_*II*_(B,IgE _*C*_,D)	k _*f*1_
	*T*_*II*_(IgE _*B*_,C,D) + IgE →*T*_*II*_(IgE _*B*_,IgE _*C*_,D)	k _*f*2_
	*T*_*II*_(B,C,IgE _*D*_) + IgE →*T*_*II*_(B,IgE _*C*_,IgE _*D*_)	k _*f*2_
	*T*_*II*_(IgE _*B*_,C,IgE _*D*_) + IgE →*T*_*II*_(IgE _*B*_,IgE _*C*_,IgE _*D*_)	k _*f*2_
D	*T*_*II*_(C,D,E) + IgE →*T*_*II*_(C,IgE _*D*_,E)	k _*f*1_
	*T*_*II*_(IgE _*C*_,D,E) + IgE →*T*_*II*_(IgE _*C*_,IgE _*D*_,E)	k _*f*2_
	*T*_*II*_(C,D,IgE _*E*_) + IgE →*T*_*II*_(C,IgE _*D*_,IgE _*E*_)	k _*f*2_
	*T*_*II*_(IgE _*C*_,D,IgE _*E*_) + IgE →*T*_*II*_(IgE _*C*_,IgE _*D*_,IgE _*E*_)	k _*f*2_
E	*T*_*II*_(D,E,F) + IgE →*T*_*II*_(D,IgE _*E*_,F)	k _*f*1_
	*T*_*II*_(IgE _*D*_,E,F) + IgE →*T*_*II*_(IgE _*D*_,IgE _*E*_,F)	k _*f*2_
	*T*_*II*_(D,E,IgE _*F*_) + IgE →*T*_*II*_(D,IgE _*E*_,IgE _*F*_)	k _*f*2_
	*T*_*II*_(IgE _*D*_,E,IgE _*F*_) + IgE →*T*_*II*_(IgE _*D*_,IgE _*E*_,IgE _*F*_)	k _*f*2_
F	*T*_*II*_(E,F) + IgE →*T*_*II*_(E,IgE _*F*_)	k _*f*1_
	*T*_*II*_(IgE _*E*_,F) + IgE →*T*_*II*_(IgE _*E*_,IgE _*F*_)	k _*f*2_

The forward rate constant *k*_*f*2_ and the cutoff distance range were optimized for each of the three energy-minimized conformations of Pen a 1 at 0 % polygon reduction. Table 5 displays the rate constants, cutoff distances, and *σ* values for the native, S-shaped, and U-shaped Pen a 1 conformations.

**Table 5:** Rule-based model parameters for three pen a 1 conformations

Parameter	Native	S-shaped	U-shaped
Cutoff distance (nm)	7.0–8.7	6.8–8.3	6.8–8.6
*k*_*f*1_ (molecule ^−1^ *s* ^−1^)	1.00	1.00	1.00
*k*_*f*2_ (molecule ^−1^ *s* ^−1^)	0.006595	0.003558	0.007315
*k*_*r*_ (s ^−1^)	0.01	0.01	0.01
*σ*	0.000703	0.000135	0.000469

**Fig. 6:**
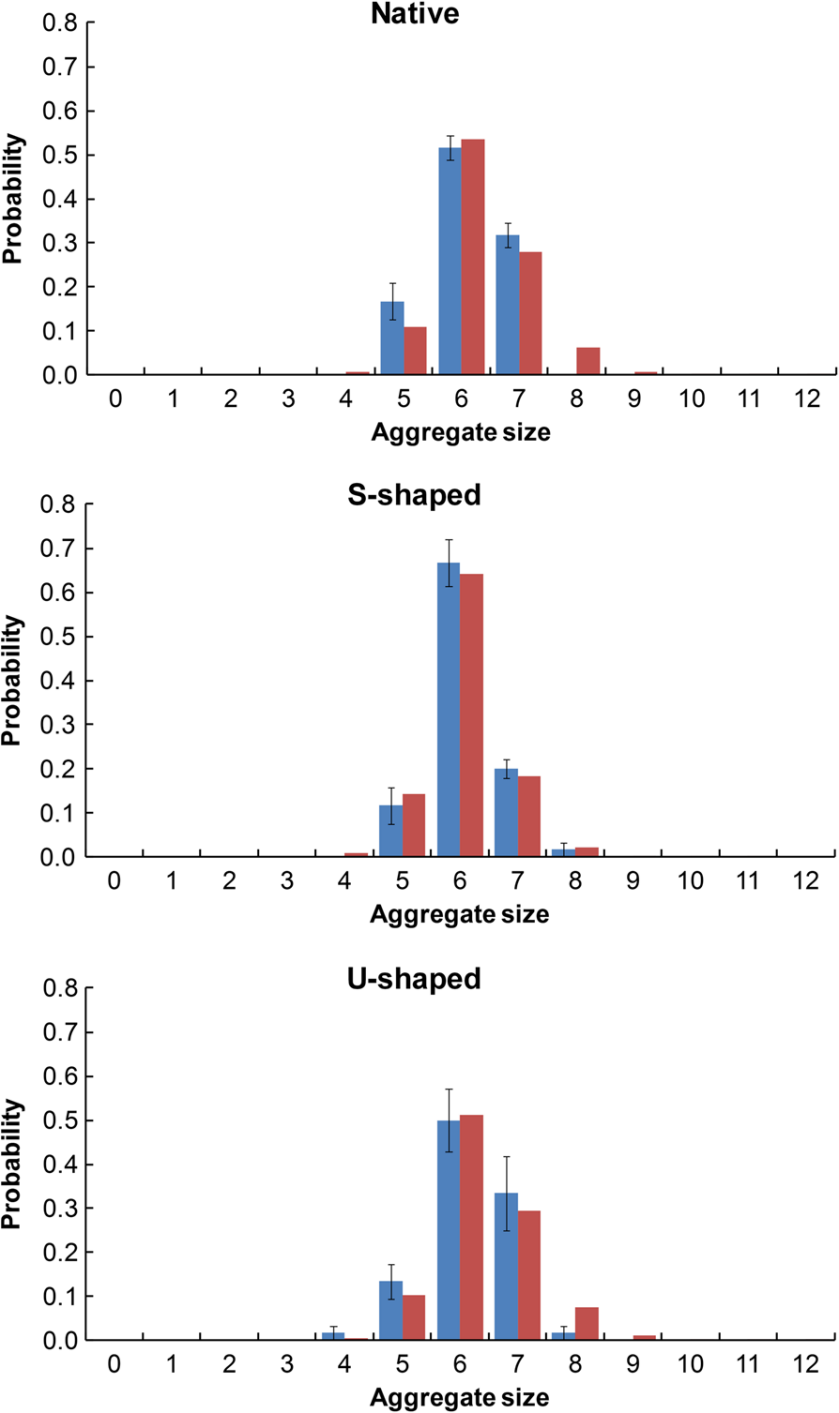
Comparison of Monte Carlo (*blue*) and rule-based model (*red*) aggregate size distributions. Results for the native (*top*), S-shaped (*center*), and U-shaped (*bottom*) Pen a 1 conformations are shown

The Monte Carlo aggregate size probability histogram data is shown along with the optimized rule-based modeling data for the native, S-shaped, and U-shaped Pen a 1 conformations at 0 % polygon reduction (Fig. 6). The error bars for the Monte Carlo data were calculated by binning the 60 runs into 10 sets of six runs each and then calculating the standard error of the mean.

### Comparing aggregate formation for each Pen a 1 conformation

For the 3D Monte Carlo simulation, we observe that there is a significant difference between the aggregate size distribution of the S-shaped conformation and that of the native and U-shaped conformations. The optimal forward binding rate constant *k*_*f*2_ shows variation between conformations, particularly between the S-shaped conformation and the other two. However, the optimal set of binding rules was found to be identical for all three conformations, and the optimal cutoff distance range is similar, but not identical, for all three conformations. This indicates that the distance at or below which two binding regions exert significant steric effects on each other is not highly dependent on conformation for the three energy-minimized conformations studied.

The rate constant *k*_*f*2_ represents the probability of an IgE-receptor complex binding to a region on the Pen a 1 molecule that is under significant steric hindrance from IgE-receptor complexes that are bound to neighboring binding regions. For the S-shaped molecule, the optimized *k*_*f*2_ value is approximately half of the *k*_*f*2_ value for the other two molecular geometries, indicating that the curvature of the S-shaped molecule may be reducing the probability that a binding region can bind without being blocked by receptors bound to nearby regions. This difference indicates that molecular geometry may play an important role in antibody aggregation onto the Pen a 1 molecule and should be taken into consideration in future aggregation research.

### Results for resolution study

In addition to molecular geometry, the resolution of the 3D molecular models used in our Monte Carlo simulation also affects the aggregation data generated by the simulation. As the resolution is reduced, the volume of the 3D models is also reduced, causing a shift in aggregate size towards larger aggregates. In this study, we seek to use our novel rule-based method to better understand the effect of resolution on the steric effects between the binding regions of the Pen a 1 molecule. We look at the probability of binding for each of the binding regions and how these probabilities vary with resolution and with molecular geometry. We also look at the variation in aggregate size distribution with resolution, and we examine the effect of model resolution reduction on the two optimized parameters used in our rule-based model: the cutoff distance and the rate constant *k*_*f*2_.

#### Probabilities of binding for individual binding regions

**Fig. 7:**
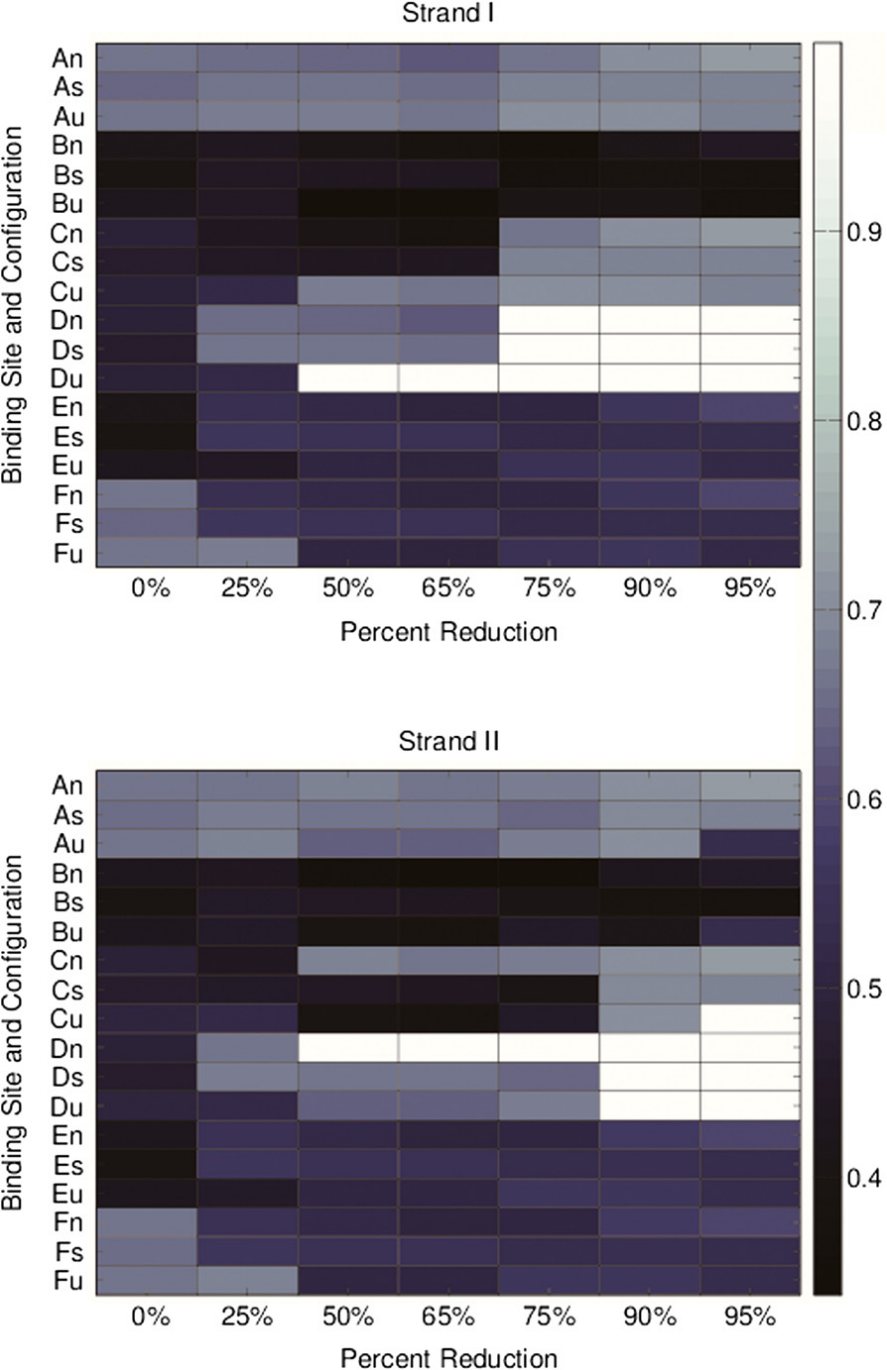
Binding probability versus resolution rule-based modeling data for the 3 Pen a 1 configurations. Data for strand I (*top*) and strand II (*bottom*) are shown. The letters A, B, C, D, E, and F represent the six binding regions used and the letters n, s, and u represent the configuration in the rule-based model. The X-axis is the percent reduction and the Y-axis are the binding sites

The probability of binding for each individual binding region of the Pen a 1 molecule varies with resolution and with conformation. For each optimized rule-based model, the probability of binding versus model resolution is shown for each of the six binding regions used in our model for the native, S-shaped, and U-shaped conformations (Fig. 7).

From the heat map we observe that region D exhibits strong variation in binding probability with resolution for both strand I and strand II for each of the three conformations. Region C exhibits strong variation for strand II of the U-shaped type. We also observe that certain pairs of binding regions display symmetry such that their binding probabilities have exactly the same value. For example, for the native type at 0 % reduced resolution, the region pairs A and F, B and E, and C and D are symmetric for both strands. This symmetry occurs due to the strong similarities between the binding rules of two or more regions; for example, the binding rules of regions in symmetric groupings encode steric effects for the same number of neighboring regions.

#### Aggregate size histograms

**Fig. 8:**
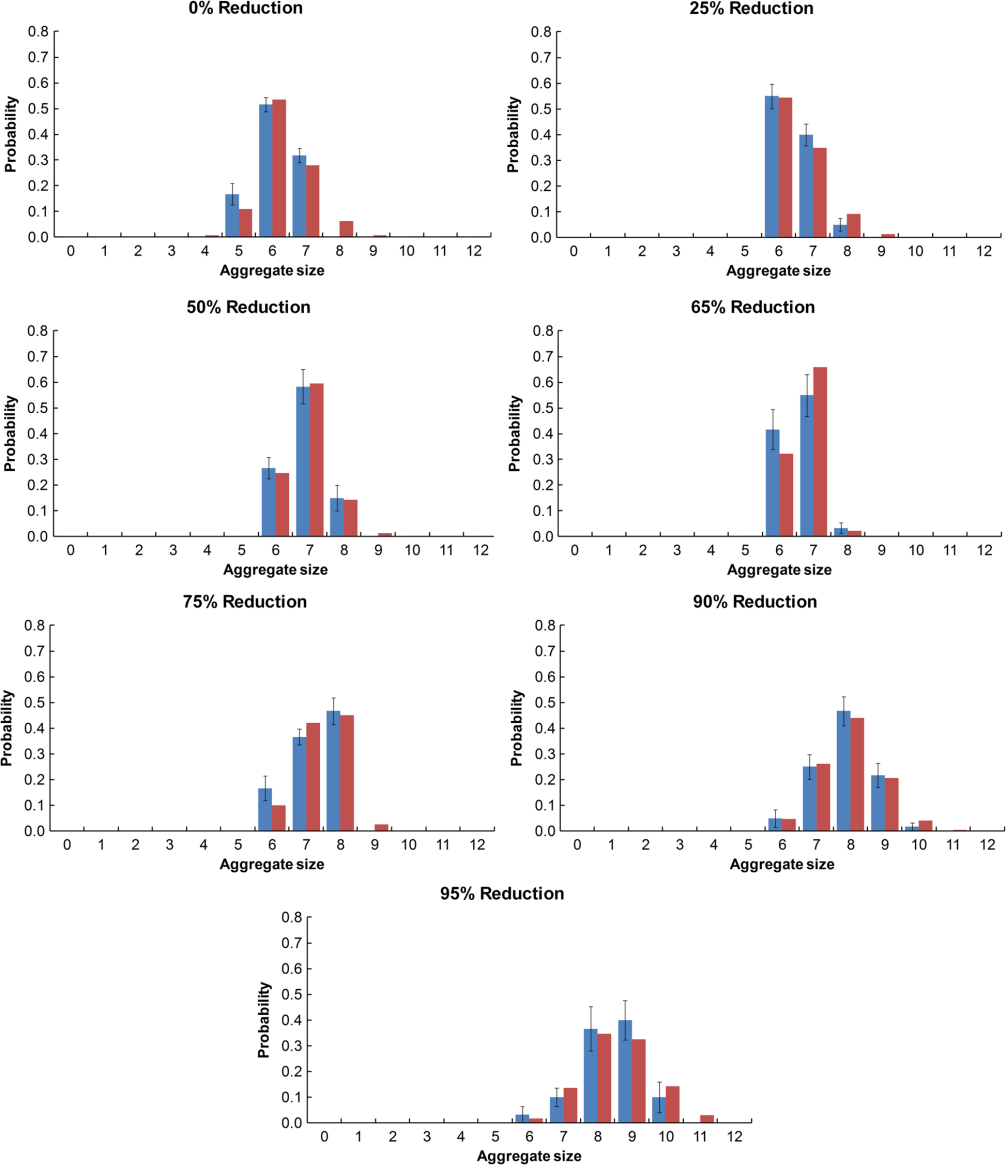
Aggregate size histograms for the native Pen a 1. A comparison of the Monte Carlo (*blue*) and optimized rule-based model (*red*) aggregate size distributions are shown. The error bars for the Monte Carlo data were calculated by dividing the 60 runs into 10 sets of six runs each and then calculating the standard error of the mean

**Fig. 9:**
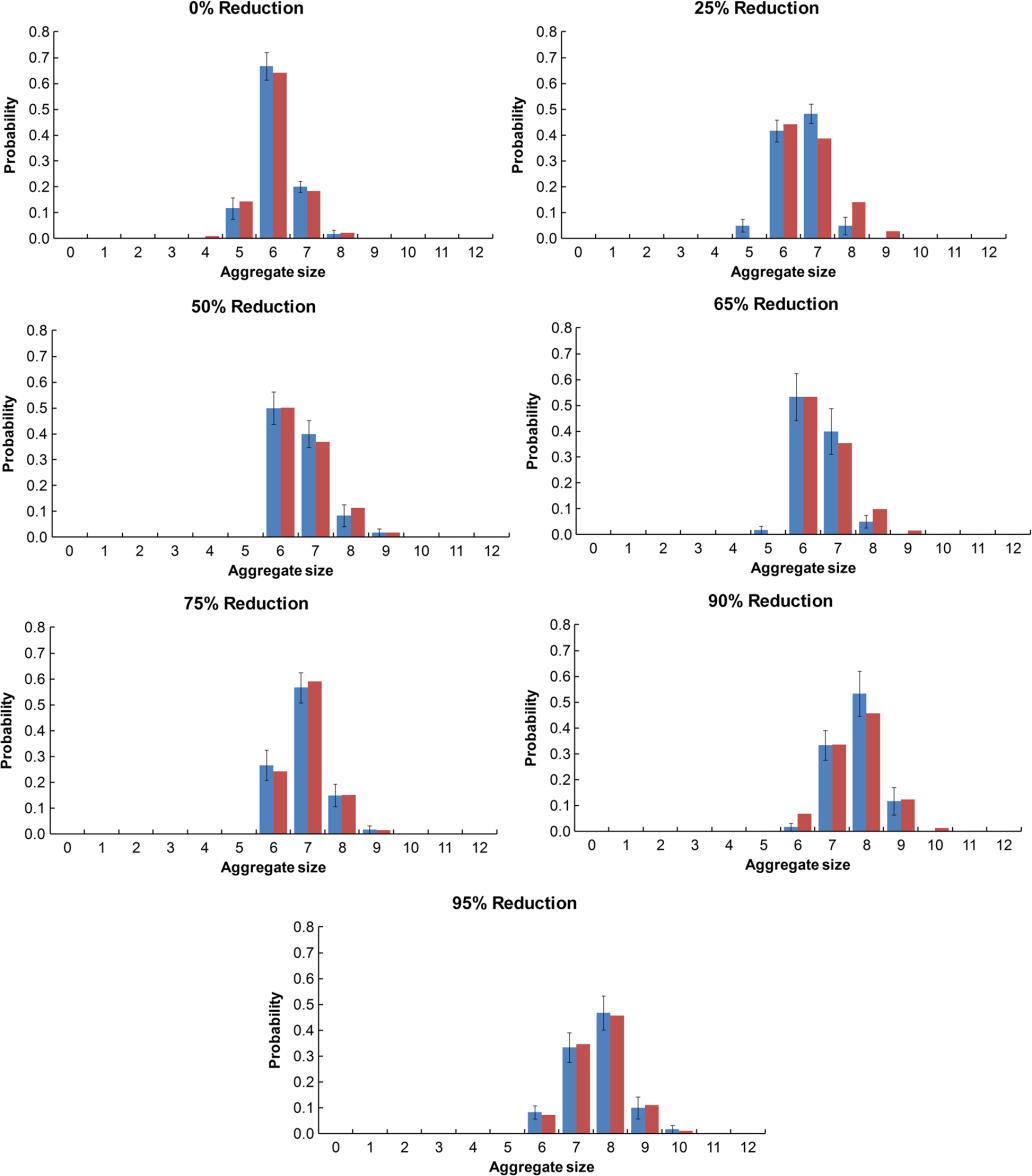
Aggregate size histograms for the S-shaped Pen a 1. A comparison of the Monte Carlo (*blue*) and optimized rule-based model (*red*) aggregate size distributions are shown. The error bars for the Monte Carlo data were calculated by dividing the 60 runs into 10 sets of six runs each and then calculating the standard error of the mean

**Fig. 10:**
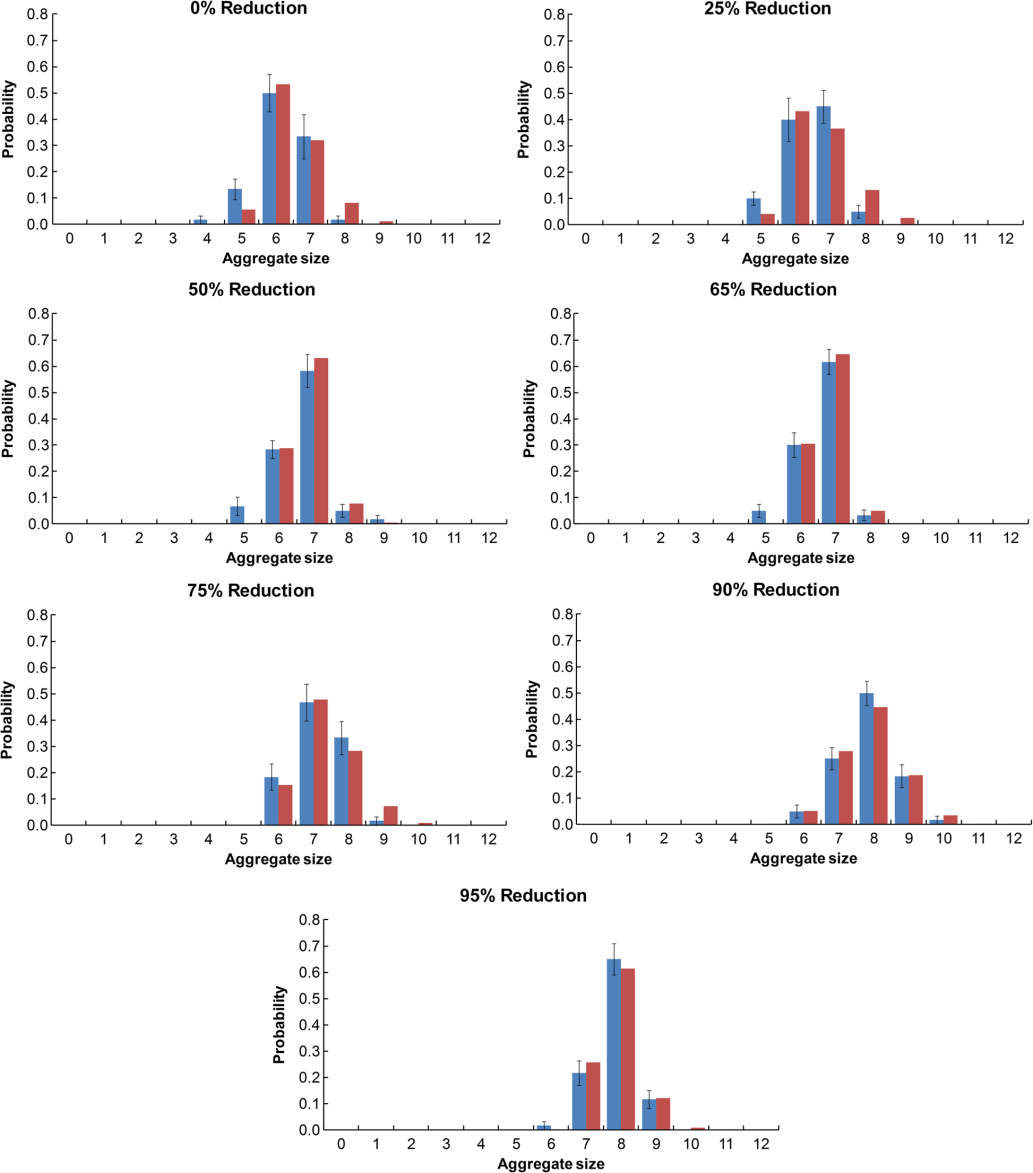
Aggregate size histograms for the U-shaped Pen a 1. A comparison of the Monte Carlo (*blue*) and optimized rule-based model (*red*) aggregate size distributions are shown. The error bars for the Monte Carlo data were calculated by dividing the 60 runs into 10 sets of six runs each and then calculating the standard error of the mean

The Monte Carlo aggregate size probability histogram data for each resolution of the Monte Carlo simulation is shown along with the optimized rule-based modeling data for the native (Fig. 8), S-shaped (Fig. 9), and U-shaped (Fig. 10) Pen a 1 conformations. The error bars for the Monte Carlo data were calculated by dividing the 60 runs into 10 sets of six runs each and then calculating the standard error of the mean. We observe that for all three conformations, the aggregate size distribution shows a general trend of shifting towards larger aggregate sizes as the resolution decreases.

#### Rate constants versus resolution

We predict that for models with the same cutoff distance range (and therefore, the same rule set), the optimized rate constant *k*_*f*2_ should increase as the aggregate size distribution shifts toward larger aggregates. This is because higher rate constants correspond with increased binding, which results in larger aggregates. Looking at the histograms in Figs. 8, 9, and 10, as well as the rate constant data in Table 6, we do indeed observe this trend, which is most clearly seen by comparing the 75 %, 90 %, and 95 % reduced resolutions of the native Pen a 1. For these resolutions, which all have the same optimized cutoff distance range, the aggregate size distribution shifts toward larger aggregates as the reduction in resolution increases, and there is a corresponding increase in the optimized *k*_*f*2_ value with reduction in resolution. We observe a similar feature for the 0 % and 25 % resolutions of the U-shaped Pen a 1.

**Table 6:** Cutoff distances (nm), binding constants (molecule ^−1^ *s* ^−1^), unbinding rate constants (s ^−1^), and *σ* values for the rule-based model for various resolutions of the native, S-shaped, and U-shaped Pen a 1

		Model percent reduction						
Cfg	Value	0 %	25 %	50 %	65 %	75 %	90 %	95 %
N	Cutoff	7.0–8.7	5.6–6.2	5.5	5.5	4.0–5.4	4.0–5.4	4.0–5.4
	*k*_*f*1_	1.00	1.00	1.00	1.00	1.00	1.00	1.00
	*k*_*f*2_	6.60e–03	7.58e–03	3.86e–03	4.90e–04	1.27e–03	1.17e–02	1.64e–02
	*k*_*r*_	0.01	0.01	0.01	0.01	0.01	0.01	0.01
	*σ*	7.03e–04	3.62e–04	6.30e–05	1.62e–03	6.31e–04	1.29e–04	9.25e–04
S	Cutoff	6.8–8.3	5.8–6.0	5.8–6.0	5.8–6.0	5.4–5.7	4.5–5.3	4.5–5.3
	*k*_*f*1_	1.00	1.00	1.00	1.00	1.00	1.00	1.00
	*k*_*f*2_	3.56e–03	1.03e–02	8.67e–03	7.84e–03	4.12e–03	6.38e–03	5.58e–03
	*k*_*r*_	0.01	0.01	0.01	0.01	0.01	0.01	0.01
	*σ*	1.35e–04	1.65e–03	1.48e–04	3.78e–04	9.40e–05	6.77e–04	4.00e–05
U	Cutoff	6.8-8.6	6.8–8.6	5.3	5.3	5.3	4.1–5.2	3.9–4.0
	*k*_*f*1_	1.00	1.00	1.00	1.00	1.00	1.00	1.00
	*k*_*f*2_	7.32e-03	1.07e–02	1.94e–03	1.18e–03	1.01e–02	1.04e–02	4.26e–03
	*k*_*r*_	0.01	0.01	0.01	0.01	0.01	0.01	0.01
	*σ*	4.69e–04	1.41e–03	5.97e–04	2.77e–04	5.23e–04	3.18e–04	2.59e–04

It may be expected that this increase in *k*_*f*2_ with a reduction in resolution should also hold true for other resolutions with the same rule set, such as the 50 % and 65 % reduced resolutions of the native Pen a 1. However, looking at the histograms in Fig. 8, we see that the Monte Carlo aggregate size distribution of the 65 % reduced resolution is shifted toward smaller aggregate sizes than is the 50 % reduced resolution. Given this data, the fact that *k*_*f*2_ is larger for the 50 % reduced resolution than for the 65 % reduced resolution makes sense. It should be noted that this unexpected feature of the Monte Carlo data may be due to the rather small number of runs (60) performed for each Monte Carlo resolution. We observe a similar feature for the 25 %, 50 %, and 65 % resolutions of the S-shaped Pen a 1, for the 90 % and 95 % resolutions of the S-shaped Pen a 1, and for the 50 % and 65 % resolutions of the U-shaped Pen a 1.

#### Cutoff distance versus resolution

**Fig. 11:**
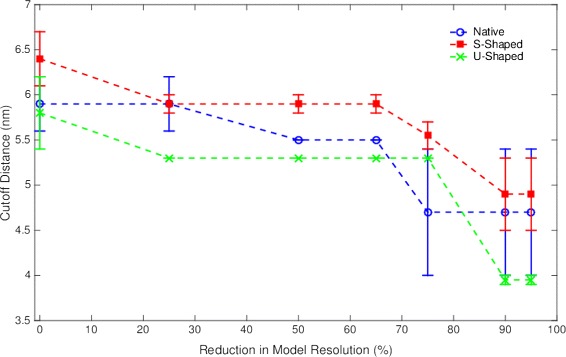
Cutoff distance versus resolution data for the 3 Pen a 1 conformations. The error bars on some of the data points represent the range of possible cutoff distances that includes the optimal cutoff distance

For each Pen a 1 conformation, we plotted the optimal cutoff distance (the cutoff distance for the rule-based model that best fit the Monte Carlo data) versus the Monte Carlo resolution for the native, S-shaped, and U-shaped Pen a 1 conformations (Fig. 11). The goal of this model is to aid in understanding how the optimal cutoff distance, and hence, the steric hindrances between binding regions vary with the resolution for different conformations of Pen a 1. The error bars on some of the data points represent the range of possible cutoff distances that correspond to the same set of rules.

As the resolution of the Monte Carlo model decreases, the volume of the molecular models decreases, and consequently the steric hindrance between binding regions decreases, resulting in a shift toward larger aggregates when there is a reduction in model resolution. This is observed in Fig. 11 for the cutoff distance range, used in this method as a measure of average steric hindrance between binding regions, which generally decreases as the resolution decreases. We also observe that some of the resolutions have the same optimized cutoff distance range. For example, the 75 %, 90 %, and 95 % resolutions of the native Pen a 1 all have a cutoff distance range of 4.0–5.4 nm.

Studying how the cutoff distance range changes with resolution provides us with useful information about how the average steric hindrance changes (or does not change) with resolution. For example, from the native Pen a 1 data, we can infer that the average steric hindrance is about the same for the 50 % and 65 % resolutions. It should be noted that some cutoff distance ranges are rather large, such as the range of 4.0–5.4 nm, and the exact optimized cutoff distance lies somewhere within that range. Therefore, for the 75 %, 90 %, and 95 % resolutions, we cannot assume that the average steric hindrance is exactly the same.

Comparing this data with the distances between binding regions on the Pen a 1 molecule allows us to predict which pairs of binding regions we expect to exhibit the greatest amount of steric hindrance on each other for each resolution of the Monte Carlo model. The cutoff distance for the 50 % and 65 % reduced resolutions of the native type is 5.5 nm. We can predict that any pair of binding regions with this or a smaller distance between them will exhibit a significant amount of steric hindrance on each other. In this case, these pairs of binding regions on strand I would be A and B, B and C, C and D, and E and F. On strand II, however, these pairs of binding regions would be A and B, B and C, and E and F. This is due to differences in curvature between the two strands. The distance between binding regions C and D on strand I is 5.5 nm, which is the cutoff distance. On strand II, the distance between binding regions C and D is 5.6 nm, which is outside the cutoff distance range, so we can predict that C and D do not exhibit significant steric hindrance on each other on strand II.

We observe that there are significant differences between the plots of cutoff distance versus resolution for each of the three conformations (native, S-shaped, and U-shaped). This is not unexpected, as the differences in curvature among the three molecules means that the distances between binding regions also vary. For example, the resolutions at which the cutoff distance range changes are not the same for the three conformations. In addition, the cutoff distances themselves vary based on conformation. These differences indicate that molecular geometry plays an important role in antibody aggregation onto the Pen a 1 molecule and should be taken into consideration in future research.

## Conclusions

We developed a novel implementation of rule-based modeling that encodes molecular geometry into the rules and associated rate constants. We studied the effects of molecular geometry on the rule sets of three U-shaped molecules of varying curvature. We also studied three energy-minimized molecular conformations of the Pen a 1 allergen using this method combined with a 3D rigid-body Monte Carlo simulation at seven different resolutions of the 3D models. We analyzed the similarities and differences among the rule-based models for each geometry and resolution to determine how the steric effects between allergen binding regions vary with molecular geometry and model resolution.

In our study of the U-shaped molecule, we found that the degree of curvature of the antigen has a strong effect on the rule set constructed using our proposed method, with individual binding regions becoming dependent on a greater number of neighboring binding regions as the degree of curvature increases. In our study of the three energy-minimized Pen a 1 conformations, we found that our proposed method of rule set construction provides a quantification of the steric effects that affect binding site accessibility and allows us to observe which neighboring binding regions most strongly affect a particular region. Although the set of binding rules will not always be different for different antigen conformations, the binding rate constants associated with the rules provide another means of quantifying the variation in aggregate size distribution based on antigen conformation.

In our resolution study, we have shown that there is a downward trend in the optimal cutoff distance with a decrease in resolution for all three of the Pen a 1 conformations. From this finding, we can conclude that the reduction in volume that results from polygon reduction of the 3D molecular models results in increased binding site accessibility. We also observed that some resolutions of the same conformation have identical optimal cutoff distances. From this data, we can extract useful information about similarities between different resolutions. We could use this data to develop models for the prediction of full-resolution aggregate size data given only lower-resolution data combined with our novel rule-based model.

We used a rod-shaped molecule in our study, but our method for measuring distances between binding sites could be modified for use with other molecular geometries. Furthermore, our method could be extended to model experimental data in the future; analysis of probe positions in electron microscopy images allow for the estimation of receptor clustering.

The integration of molecular geometry with rule-based modeling to simulate molecular assembly processes produces detailed insights into binding site accessibility while overcoming the problem of combinatorial complexity. These geometric insights can provide quantitative information on the steric accessibility of binding regions, therefore providing details that were not traditionally produced from rule-based modeling. Another approach to gain computational efficiency, reduced resolution models, can be used to model assembly structures in reasonable time-frames. However, the impacts of reduced resolution can be difficult to quantify. We have shown, through the integration of geometric rule-based modeling and three-dimensional reduced resolution Monte Carlo simulations, that this difference can both be quantified and potentially used for evaluating the quality of reduced resolution structures.
